# Comprehensive at-home sexually transmitted and blood borne infection (STBBI) testing program: A pilot study

**DOI:** 10.1177/09564624251371793

**Published:** 2025-08-25

**Authors:** Brandon L. Christensen, Chantal L. Rytz, Jason E. Black, Grace Kwon, Nolan E. Hill, Pam Krause, Kevin Fonseca, Myles Leslie, Deirdre L. Church, Christopher T. Naugler, Phillip Lacap, John Kim, Caley B. Shukalek, Ranjani Somayaji

**Affiliations:** 1 Cumming School of Medicine, 70401 University of Calgary, Calgary, AB, Canada; 2 Department of Medicine, 70401 University of Calgary, Calgary, AB, Canada; 3 Libin Cardiovascular Institute, 70401 University of Calgary, Calgary, AB, Canada; 4 O’Brien Institute for Public Health, 70401 University of Calgary, Calgary, AB, Canada; 5 585584 Center for Sexuality, Calgary, AB, Canada; 6 Alberta Precision Laboratories, Calgary, AB, Canada; 7 Department of Pathology and Laboratory Medicine, 70401 University of Calgary, Calgary, AB, Canada; 8 School of Public Policy, 70401 University of Calgary, Calgary, AB, Canada; 9 National Laboratory for HIV Reference Service, National Microbiology Laboratory Branch, 41687 Public Health Agency of Canada, Winnipeg, MB, Canada; 10 Snyder Institute for Chronic Diseases, 70401 University of Calgary, Calgary, AB, Canada

**Keywords:** Sexually transmitted infections and blood borne infections, remote testing, surveillance, diagnosis, pilot study

## Abstract

**Background:**

Rates of sexually transmitted and blood borne infections (STBBI) are rapidly increasing. Despite the high diagnostic accuracy of self-testing, no fully remote STBBI testing programs are available in Canada. We aimed to evaluate the feasibility and acceptability of a fully-remote, web-based, at-home STBBI testing (self-collection) program in Calgary, Canada.

**Methods:**

Participants who were Alberta residents aged ≥16 years self-enrolled into a web-based platform between February 2023 and March 2024 and completed consent and intake questionnaires consisting of demographic and sexual health data. Kits were mailed, and samples were self-collected including swabs, urine and dried blood spot cards. Results of processed kits were communicated securely to participants. Surveys to assess the feasibility and acceptability of the process were completed.

**Results:**

Of the 156 participants (39.7% men, 37.3 ± 10.5 years) from diverse sex and gender backgrounds who completed the intake, 43% (*n* = 67) participants returned their testing kits. In the cohort, there was low reported condom use in more than 50%, and 40% had not had STBBI testing in the past 12 months. There were six participants (9.0%) with new positive tests for an STBBI and all were connected with appropriate treatment. Participants largely reported satisfaction with the web-based platform and testing process as well as ease with testing modalities with the except for dried blood spot testing which presented collection challenges.

**Conclusions:**

Our web-based comprehensive testing pilot was feasible and acceptable, demonstrating the value of such remote approaches to diminishing the threat of rising STBBI rates.

## Introduction

Rates of sexually transmitted and blood borne infections (STBBI) have seen a concerning rise in recent decades, with an estimated one million curable cases acquired daily.^1^ Despite global progress, stigma and discrimination continue to present significant barriers to diagnosis, treatment and prevention strategies.^2,3^ These barriers are particularly pronounced in populations with limited healthcare access, persons who use drugs, as well as racial/ethnic, and gender and sexual minorities who face disproportionately greater rates of infection, decreased testing, and poorer outcomes.^4,5^

Routine screening is integral to STBBI prevention and control though clinic-based programs may capture a limited proportion of individuals due to inaccessibility, discrimination, and discomfort discussing sexual practices with healthcare providers.^3^ In response, self-testing initiatives leveraging digital technologies have been developed outside Canada to offer a convenient alternative that consistently demonstrates accuracy and validity comparable^6^ or even superior^6,7^ to traditional laboratory-based tests. Internet-accessed STBBI testing has exhibited significant adoption in a diverse range of populations,^3,8^ greater rates of repeat testing^9^ and reduced time-to-test among ‘never testers’,^10^ underscoring the acceptability and feasibility of a remote screening program.^11^

Although STBBI self-testing has been established as a valid modality to quickly identify and prevent potential complications or transmission,^10,11^ it remains underutilized. STBBI programs have largely continued to use in-person specimen collection and/or care.^3,12^ When self-testing has been used, it has been limited in scope, either in the tests offered,^3^ or in the cohorts it is offered to (e.g., men who have sex with men [MSM]).^12,13^ This is particularly the case in Canada, which has few remote testing options available. Thus, the feasibility and accessibility of a fully remote program is not currently known. Therefore we aimed to evaluate the feasibility of a comprehensive, and fully remote STBBI care pathway. We hypothesized that the platform would be feasible, acceptable, and might reduce barriers to, STBBI testing.

## Methods

### Recruitment

We conducted a prospective cohort study between February 2023 and March 2024 and participants were recruited online or in-person at the Center for Sexuality (CFS; Calgary, Canada). The study was advertised on the CFS website and social media, the investigators’ social media, as well as the University of Calgary external study site using ethics board approved materials. All recruited participants self-enrolled online using a web-based platform (athomestitesting.ca) which included online consent and intake questionnaires which were hosted on a secure platform through REDCap (University of Calgary server). Participants were eligible if they were residents of the province of Alberta, ≥16 years of age, and able to provide written informed consent. The intake form collected information on demographics (age, sex, gender, sexual orientation), sexual health and history, STBBI history, and information on perceived barriers and stigma relating to their sexual health.

### Ethics

The study was approved by the University of Calgary Research Ethics Board (REB22-0476).

### STBBI testing

Sample collection was self-performed by participants predominantly at home but was permitted to be done by participants on site at the Center for Sexuality, if they preferred, following the completion of the intake survey. Multi-site sampling was offered for bacterial sexually transmitted infections APTIMA® multitest swabs and first-voided urine specimens (Hologic Canada, Missisauga, ONT.). Valid samples included first-catch urine and pharyngeal, vaginal or rectal swabs. Bacterial STBBI testing was limited to *C. trachomatis* (CT) and *N. gonorrhoeae* (NG). First-catch urine was transferred to the appropriate urine transport tube within 24 h to maintain sample stability. These tests were performed locally at Alberta Precision Laboratories using the APTIMA COMBO 2® @ Assay (for CT/NG) performed on a Panther® system (Hologics Canada). All molecular detection assays were strictly performed and reported according to the manufacturer’s instructions.

Sample collection for *T. pallidum* (syphilis), human immunodeficiency virus (HIV) and hepatitis C virus (HCV) were obtained using self-collected dried blood spots (DBS) via fingerstick on Protein Saver Cards that were transported to the National Microbiology Lab in Winnipeg, Canada, for testing previously using validated methods. Syphilis testing was limited to a screen only and was conducted with either the Bio-Rad BioPlex 2200 Syphilis Total assay or, if insufficient sample available, the Bio-Rad Syphilis Total Antibody enzyme immunoassay (EIA). HCV serological screening was performed using either the Abbott Alinity Anti-HCV chemiluminescent magnetic microparticle immunoassay (CMIA) or, if limited sample availability, the Ortho® HCV EIA. HCV reactive samples were reflexed to molecular confirmatory testing using the Hologic Aptima® HCV Quant assay for RNA viral load quantification. Similarly, HIV serological screening was performed using the AbbottAlinity® HIV antigen/antibody combo assay (CMIA) or, if limited sample availability, the Avioq HIV-1 Microelisa assay (EIA). Molecular confirmatory testing for HIV reactive samples was performed with the Hologic® Aptima HIV-1 Quant assay for RNA viral load quantification. High and durable sensitivity and specificity has previously been demonstrated using DBS for both HIV^14^ and HCV.^15^

### Test kit creation and sample collection

Details from the intake questionnaire around sexual practices including the types and sites of sexual activity informed the creation of a personalized, behaviour-based testing kit for each participant. Testing kits were mailed the next business day to participants including test collection instructions, links to instructional videos, testing materials (e.g., swabs), contact information for the study team, and a pre-paid return envelope. All test kits and materials were pre-labeled with the participants unique de-identified participant identification number. After completion, participants securely mailed their self-collection kits back to the study team. Test kits were processed Monday to Friday and samples were forwarded to the appropriate laboratory for testing, as outlined above.

### Testing results and communication

Tracking of sent and pre-paid return envelopes was monitored to ensure delivery. Results were communicated electronically both from the Alberta Precision Laboratory and National Microbiological Laboratory to the study team who entered them into a secure database. Upon enrollment, participants selected whether they wished to receive results via secure SMS or by email and this contact information was validated via confirmation code. Notification of negative results was automatically generated by REDcap to participants. The electronic health record was accessed to determine if any positive tests (in particular for HIV/HCV/syphilis) were new cases prior to communicating the results. Participants with positive or inconclusive results were manually contacted for disclosure. Links to treatment or care were provided by the study team where positives were identified.

### Testing experiences

Two short anonymous electronic surveys were used to evaluate the testing process. The first was sent immediately after test kit return evaluating difficulties with the kit or collection process. The second was sent after all results had returned evaluating the ease of each collection method, communication, and online testing process overall. Unstructured qualitative responses were solicited for both surveys.

### Data collection

All data were stored in existing secure computing environments (University of Calgary) with strict security measures in alignment with Alberta Health Services policies and procedures. Data was de-identified prior to analysis and was analyzed in aggregate. All team members who had direct access to data for care decisions were clinicians with STBBI expertise licensed to practice in Alberta.

### Statistical analyses

The primary outcomes of the pilot study were the feasibility and acceptability of the online testing platform and testing kits. We defined feasibility and acceptability a priori (goal 80%) as the proportion of participants who were able to receive test results following completion of the survey and self-collection (i.e., those who returned the kits) and rated it as satisfactory. Additional outcomes included testing and treatment turnaround times along with test kit return rates. Free text qualitative responses relating to the process were also collected synthesized from participants survey responses. Baseline characteristics were summarized for all respondents and the subset who returned their test kit using frequencies and proportions for categorical variables. Means and standard deviation or medians and interquartile ranges were used for continuous variables, as appropriate. Analysis to explore differences in age, gender identity and sexual orientation were completed amongst individuals who did and did not return their kits using Students unpaired *t* test, Chi squared tests, and Fischer’s exact tests, as appropriate. Among respondents who returned the testing kit, various STBBI testing parameters (i.e., kit return and communication times, tests performed, and positive test results) and STBBI self-sampling experiences of respondents (i.e., satisfaction, ease, communication, and challenges) were characterized using frequencies and proportions, means and standard deviations, and medians and interquartile ranges. All quantitative analyses were conducted through R 4.0.4^©^. Unstructured qualitative responses to the surveys were analyzed inductively to identify key themes. We present a representative selection of these data below, sorted according to the themes of ‘negative’ and ‘positive’ feedback.

## Results

**Figure 1.**
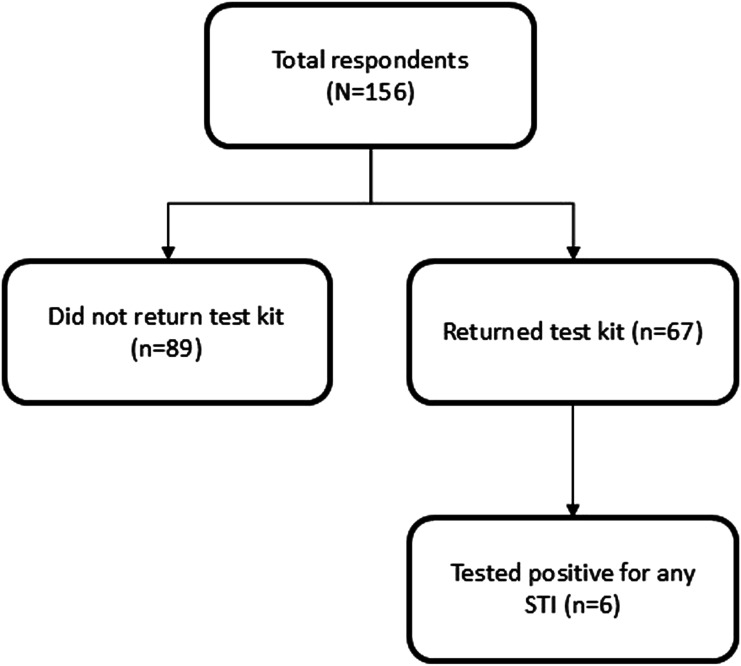
Study flow diagram.

A total of 156 participants enrolled during the study period and three individuals used the program more than once. Of the cohort, 62 were men (39.7%), the mean age was 37.3 years (SD 10.5), and the majority identified as members of the LGBTQ2+ community (Table 1). Of those who completed the intake survey and received a kit, 67 (43%) completed self-collection and returned it (Figure 1). The demographics (age, gender identity) were similar between those who did and did not return the kits, but there were some differences noted amongst gender identities, with fewer gender-fluid, non-binary/non-conforming, queer or two-spirit individuals returning the kit. In the study, 16 participants picked up the testing kit on site at the CFS, and compared to the full cohort, they tended to be older and more likely to identify as men. Although the study was targeted to the Calgary region (based on shipping logistics), two participated from rural areas of Alberta and some participated from Edmonton, Lethbridge, and other sites (self or parent’s address). Otherwise, the geographic distribution was relatively even between the quadrants (NE-23 (20.5%); NW-32 (28.6%); SE-19 (17.0%); SW-27 (24.1%)) for those in Calgary.

**Table 1.** Baseline characteristics of cohort.

	Total cohort (*N* = 156)	Respondents who returned test kit (*n* = 67)
Age (years), mean (SD)	37.3 (10.5)	37.8 (10.1)
Gender, *n* (%)		
Woman	53 (34)	26 (38.8)
Man	62 (39.7)	31 (46.3)
Gender-fluid, non-binary/non-conforming, queer or two-spirit	38 (24.4)	10 (14.9)
Self-describe/prefer not to answer	3 (1.9)	0
Identity, *n* (%)		
Cisgender	120 (76.9)	54 (80.6)
Transgender	18 (11.5)	5 (7.5)
Self-describe	3 (1.9)	2 (3)
No response	15 (9.6)	6 (9)
Sexuality*, *n* (%)		
Straight	22 (14.1)	11 (16.4)
Heterosexual	20 (12.8)	11 (16.4)
Homosexual	14 (9)	7 (10.4)
Gay	33 (21.2)	16 (23.9)
Lesbian	4 (2.6)	1 (1.5)
Bisexual	36 (23.1)	14 (20.9)
Pansexual	26 (16.7)	9 (13.4)
Queer	30 (19.2)	10 (14.9)
Self-describe/prefer not to answer	10 (6.4)	4 (6)
STBBI in the last year?, *n* (%)		
None	125 (80.1)	54 (80.6)
One or more	22 (14.1)	9 (13.4)
No response	9 (5.8)	4 (6)
Time since last STBBI test, *n* (%)		
No prior testing	16 (10.3)	5 (7.5)
<1 month	11 (7.1)	4 (6)
1–3 months	28 (17.9)	10 (14.9)
4–6 months	22 (14.1)	13 (19.4)
6–12 months	30 (19.2)	15 (22.4)
>12 months	47 (30.1)	20 (29.9)
No response	2 (1.3)	0
Relationship status, *n* (%)		
Single	60 (38.5)	24 (35.8)
Monogamous	20 (12.8)	13 (19.4)
Open	69 (44.2)	28 (41.8)
Prefer not to answer/Self describe	5 (3.2)	2 (3)
No response	2 (1.3)	0
Number of partners in the last 6 months?, *n* (%)		
0	6 (3.8)	3 (4.5)
1–5	110 (70.5)	47 (70.1)
6–10	19 (12.2)	12 (17.9)
>10	17 (10.9)	5 (7.5)
No response	4 (2.6)	0
Types of sex in the last 6 months, *n* (%)		
Oral	145 (92.9)	63 (94)
Vaginal	103 (66)	42 (62.7)
Anal provided	70 (44.9)	26 (38.8)
Anal received	68 (43.6)	37 (55.2)
Condom use in the last 6 months, *n* (%)		
0%	50 (32.1)	24 (35.8)
<50%	37 (23.7)	10 (14.9)
Approx. 50%	14 (9)	6 (9)
>50%	20 (12.8)	9 (13.4)
100%	28 (17.9)	16 (23.9)
No response	7 (4.5)	2 (3)
Substance use in the last 6 months, *n* (%)		
Alcohol	72 (46.2)	31 (46.3)
Cannabis	67 (42.9)	27 (40.3)
Other drugs/substances	12 (7.7)	5 (7.5)
No response	5 (3.2)	1 (1.5)
Has primary care provider?, *n* (%)		
Yes	100 (64.1)	48 (71.6)
No	54 (34.6)	19 (28.4)
No response	2 (1.3)	0
Comfort discussing sexual health needs with provider^a^, *n* (%)		
Extremely uncomfortable	11 (11)	2 (4.2)
Somewhat uncomfortable	29 (29)	13 (27.1)
Neither comfortable nor uncomfortable	10 (10)	6 (12.5)
Somewhat comfortable	22 (22)	16 (33.3)
Extremely comfortable	27 (27)	11 (22.9)
No response	1 (1)	0
How satisfied are you with your current level of access to sexual health services (e.g., STBBI testing, counselling, treatment, education)?, *n* (%)		
Extremely dissatisfied	31 (19.9)	9 (13.4)
Somewhat dissatisfied	42 (26.9)	19 (28.4)
Neither satisfied nor dissatisfied	31 (19.9)	13 (19.4)
Somewhat satisfied	37 (23.7)	22 (32.8)
Extremely satisfied	12 (7.7)	4 (6)
No response	3 (1.9)	0

^a^Among those with a primary care provider.

Age and identity were similar between those who returned the kit and those who did not (p = .59 and p = .28, respectively); however, some gender differences existed (p = .03) with fewer gender-fluid, non-binary/non-conforming, queer or two-spirit individuals returning the kit.

Slightly less than half of respondents returned the testing kit (*n* = 67; 42.9%).

Among those who enrolled, most were single (38.5%) or in an open relationship (44.2%), and 70.5% reported having 1–5 partners in the past year (with 10.9% reporting >10 partners). Condom use was reported as <50% of the time by more than half of the cohort (55.8%), and nearly one-third (32.1%) reported zero use. Twenty-two participants (14.1%) reported having an STBBI in the last year, but 63 participants (40.4%) reported they had either had no prior STBBI testing or had not had testing for >12 months. In participants who reported having a primary care provider, 40% were somewhat or extremely uncomfortable discussing their sexual health needs. In terms of access to STBBI services, 46.8% were somewhat or extremely dissatisfied while 31.4% were somewhat or extremely satisfied (Table 1).

In the 67 participants who returned kits, the median time was 11 days (IQR 7–27). Four participants (6%) who returned the kits did not complete DBS testing. In one case, the DBS test had insufficient sample to process so another test was sent to the participant to repeat this. Results were provided for CT/NG at a median time of 6 days (IQR 4–10.8) and for HIV/Syphilis/HCV in 29 days (IQR 23–37). Five participants (7.5%) had a positive test for CT/NG and six (9.0%) tested positive for HIV/HCV/syphilis. Five of the latter participants had previous positives with three previously positive for HIV (4.5%), two for syphilis (3.0%) and one for HCV (1.5%). One participant of this group was co-infected with HIV and syphilis. One participant (1.4%) had a new positive for syphilis based on DBS testing (Table 2). Participants who tested positive for an STBBI (*n* = 6; 9.0%) were similar in age (37.1 years, SD 14.0) to the total cohort, but more likely to identify as men (66.7%). All participants who had new infections were connected with a community-based facility for treatment (Table 3).

**Table 2.** STBBI testing parameters and results.

	Test kits returned (*n* = 67)
Time to return kit return (days), median (IQR)	11 (7–27)
Tests performed, *n* (%)	
CT urine	29 (43.3)
CT vagina	34 (50.7)
CT throat	62 (92.5)
CT rectal	40 (59.7)
NG urine	30 (44.8)
NG vagina	33 (49.3)
NG throat	62 (92.5)
NG rectal	41 (61.2)
HIV DBS	57 (85.1)
Syphilis DBS	63 (94)
HCV DBS	60 (89.6)
Time from test kit return to results communication GC/CT (days), median (IQR)	6 (4–10.8)
Time from test kit return to results communication HIV, HCV, Syph (days), median (IQR)	29 (23–37)
Any positive GC/NG test, *n* (%)	5 (7.5)
Any positive HIV/Syphilis/HCV test, *n* (%)	6 (9.0)
Any positive HIV/Syphilis/HCV test (excluding previous positives), *n* (%)	1 (1.4)

**Table 3.** Participant survey of STBBI testing.

	Respondents who returned test kit (*n* = 67)
Satisfaction with online testing (1 = extremely dissatisfied; 5 = extremely satisfied), median (IQR)	4 (4–5)
How difficult was each test (1 = very difficult; 7 = very easy), median (IQR)	
Swab	7 (6–7)
Urine test	7 (6–7)
Dried blood	5 (3–6)
How satisfied were you with the communication of results? (1 = not satisfied at all; 7 = very satisfied), median (IQR)	6 (5–7)
How likely would you be to use this online testing service again? (1 = not likely at all; 7 = very likely), median (IQR)	7 (6–7)
Did you experience any issues with the following? (yes), *n* (%)	
Swab collection	2 (3)
Urine collection	2 (3)
Dried blood spot	25 (37.3)
Accessing or using technology	2 (3)
Instructions for testing process	9 (13.4)
Where did you get treatment?^a^, *n* (%)	
Community pharmacy	1 (16.7)
PCP	1 (16.7)
Sexual health clinic	1 (16.7)
Emergency or urgent care	0
Not treated yet	0
Other	0
No response	3 (50)

^a^Among those who tested positive for at least one STI.

Participants who completed testing (*n* = 67) reported that they were satisfied with the online platform (median score 4; IQR 4–5). They expressed satisfaction with the communication of results, and stated that they would be very likely to use the service again (median 7; IQR 6–7) (Table 3). Participants reported that the easiest tests were the bacterial swab and urine sampling (median 7; IQR 6–7). The most issues experienced were with DBS testing (37.3%), whereas there were minimal issues identified with other sample collections, or use of technology (online platform).

Analysis of the unstructured qualitative feedback identified both positive and negative themes. Negatively, participants expressed concerns primarily about difficulties they encountered with DBS testing, notably with obtaining sufficient blood to fill the card circle. Positive comments focused on the testing platform, and there were individual comments relating to labelling with addresses and personal information, and communication of results. An illustrative selection of these comments follows:

### Negative feedback

Dried blood spot:

 o *“I think one more lancet would’ve been helpful for the blood collection process. I used both but still couldn’t fill up all of the circles”*
 o *“The biggest issue was trying to get the blood sample as the drops of blood were not enough on their own to fill one circle”*
 o *“It was really not clear that I had to twist off the purple part of the lancet; I only figured it out after looking online”*

Other:

 o *“The QR code for the testing instructions did not work for me”*
 o *“I was unsure whether I should have included my name and collection date on the collection tubes”*

### Positive feedback

Kit and process:

 o *“Extremely easy process. Great instructions and easy to mail with the prepaid envelope.”*
 o *“I’d much rather do this than go to a clinic”*
 o *“I’m just so happy something like this exists! It removes the barrier to get tested and thank you for putting this together!”*

## Discussion

Our prospective study of a fully online comprehensive STBBI self-sampling platform in a large Canadian health care region demonstrated feasibility and acceptability. Collaboration with a sexual health center focused on caring for persons identifying as members of the LGBTQ2S + community and an online platform enabled participation from a more gender- and sexually-diverse group. A significant proportion of participants had not undergone STBBI testing in 12 more months, reported limited to no condom use, and did not have ready access to a primary care provider (34.6%). Those who completed self-collection had their tests processed and results communicated, and all persons with new infections were connected with appropriate treatment demonstrating feasibility of the fully remote (online) platform. Importantly, participants who completed the process reported satisfaction with the testing and results notification and would be likely to use the platform again.

In our pilot remote STBBI platform, we offered participants the ability to receive mailed kits or pick them up at a sexual health center. Of the 156 participants who took the intake survey, 67 (43%) completed and returned the testing kits. Return rates of people who completed the intake survey were consistent with another pilot study conducted in Vancouver as well as a multi-site evaluation in the United States which had rates of 37%–46%.^16,17^ The positivity rates in our cohort were similar to that of a statewide US study^17^ (9.0% vs 9.6%), but were more similar to the in-person testing rates versus lower rates from an online Canadian cohort study.^3^ Notably, there were differences with fewer STBBI pathogens tested than in our present study. As there are increased detection rates when extra-genital testing is offered,^18^ it is possible that our comprehensive testing strategy may have led to the increased positivity rates.

The integration of novel at-home STBBI testing and care delivery measures is increasingly accepted by both service providers^19^ and patients^20^ and may enhance uptake across numerous subgroups.^8^ More than 40% of the participants in our study reported pre-existing discomfort or dissatisfaction with discussing their sexual health needs and low access to STBBI services. Conversely, participants largely reported satisfaction with our online STBBI platform and process, quantitatively declaring they would likely use the services again (median score 7 (IQR 6–7)) and also providing positive qualitative feedback. In terms of testing, ease was reported with the bacterial swabs and urine sampling, whereas some participants experienced challenges with DBS testing. A study evaluating the feasibility of home collected samples using DBS for HIV similarly identified a degree of dissatisfaction for collecting blood by fingerstick rather than standard venipuncture.^21^ Another study of DBS testing for syphilis, HIV and Hepatitis C/B identified that although it was acceptable, collecting sufficient blood for the cards was a challenge as similarly expressed by participants in our cohort, and sensitivities for syphilis and hepatitis C in their cohort were low.^22^ These findings highlight the need to consider further innovation of the fingerstick modality kit (i.e., longer lancet) or alternative sampling methods to improve acceptability and test characteristics as well as exploration of preferences of fingerstick and conventional lab-based tests.

As one of the first fully online comprehensive STBBI platforms to be piloted in a large Canadian health region, strengths and limitations must be considered. We used a behaviour-based multisite testing approach with swabs, fluid and DBS enabling comprehensive target testing with the potential for enhanced detection.^18,23^ We successfully engaged a diverse range of participants including those from gender and sexual minorities and had a feasible process with a relatively fast timeframe from receipt of tests to results even accounting for shipments of DBS testing to the National Microbiology Laboratory. The acceptability and satisfaction of the online process was expressed by the participants, and included requests for an expanded launch of such an initiative. Concurrent to our pilot, a province-wide initiative was launched using our study findings as foundational pilot data, but the initiative unfortunately was cancelled based on changes to the health system priorities during the time period. Nonetheless, given the significant and real concerns of stigma as pertains to sexual health, especially in many minority groups and in geographically distant areas, remote testing can serve at minimum as an important adjunct process for STBBI care.

Regarding limitations of our study, our study participants were healthy and had access to technology (phone, internet) and our sample size was smaller, thereby limiting the interpretability and generalizability of our findings. However, we had a diverse socio-demographic group with no major differences between those who did and did not complete testing, and had a number of positive cases (7.5%) in what was likely to be an asymptomatic cohort, leading to increased confidence in our results. Although less than half of the participants completed testing and returned the kits, this was similar to other studies, and we still demonstrated feasibility and acceptability of the process. The DBS test was noted to be challenging including by way of qualitative feedback, but the vast majority of those who returned the kits were able to still successfully complete the test. We did not include STBBI education along with our platform and testing process, but given the limited testing frequency and condom use as reported by the participants, additional education and resources will be an important aspect to incorporate into any online STBBI testing program.

Our home-collected, completely remote multi-site, behaviour-based screening program proved to be both highly acceptable (80%) and feasible across a large healthcare region, including the ability to identify numerous potential care deficits in populations of rarely or never-tested individuals. Therefore, these findings underscore the importance of implementing such programs to minimize healthcare disparities and improve access to STBBI testing and care for underserved populations.

## Conclusion

Among participants in a large Canadian province, our fully at-home STBBI self-sampling platform pilot was feasible, and reported to be acceptable by participants from a diverse background of sexual and gender groups. Use of online testing processes may be a valuable adjunctive strategy to improve STBBI testing while mitigating perceived stigma and discrimination to address the growing burden of STBBI in Canada.
